# Evolution of island lizards remains a mystery

**DOI:** 10.7554/eLife.62230

**Published:** 2020-09-22

**Authors:** Kathryn D Kavanagh

**Affiliations:** Department of Biology, University of Massachusetts DartmouthNorth DartmouthUnited States

**Keywords:** Anolis sp., evolution, plasticity, adaptation, morphometrics, developmental bias, Other

## Abstract

Lizards that live in the Greater Antilles exploit a large range of skeletal variations to adapt to similar habitats, in defiance of the theory of plasticity-led evolution.

**Related research article** Feiner N, Jackson IS, Munch KL, Radersma R, Uller T. 2020. Plasticity and evolutionary convergence in the locomotor skeleton of Greater Antillean *Anolis* lizards. *eLife* **9**:e57468. doi: 10.7554/eLife.57468

The effects of mechanical stress on the bones of baby animals might not come to mind when you think about evolution, but recent theoretical work suggests that there might be a connection between the two. Environmental stresses can lead to small variations amongst developing embryos that natural selection can act upon, and recent research suggests that the way that variations are produced during development may guide the evolutionary paths that lineages follow (Brun-Usan et al., 2020; Uller et al., 2020; Levis and Pfennig, 2016; Moczek et al., 2011).

Developmental plasticity refers to how embryonic development responds to the environment: in particular, it refers to the way that an individual genotype interacts with its environment during development to produce a particular phenotype. The idea is that over many generations, this interaction with the environment can itself be 'tuned', so embryos consistently produce the right phenotypes for the environments they might encounter. The downside of such tuning is that some variations disappear, which means that the species cannot evolve in certain directions. Tuning therefore makes evolution more predictable and less exploratory (Figure 1). Plasticity-led evolution seems like a good way to jump-start adaptation to changing environments, but so far, this idea has been mostly theoretical, with few real examples.

**Figure 1.**
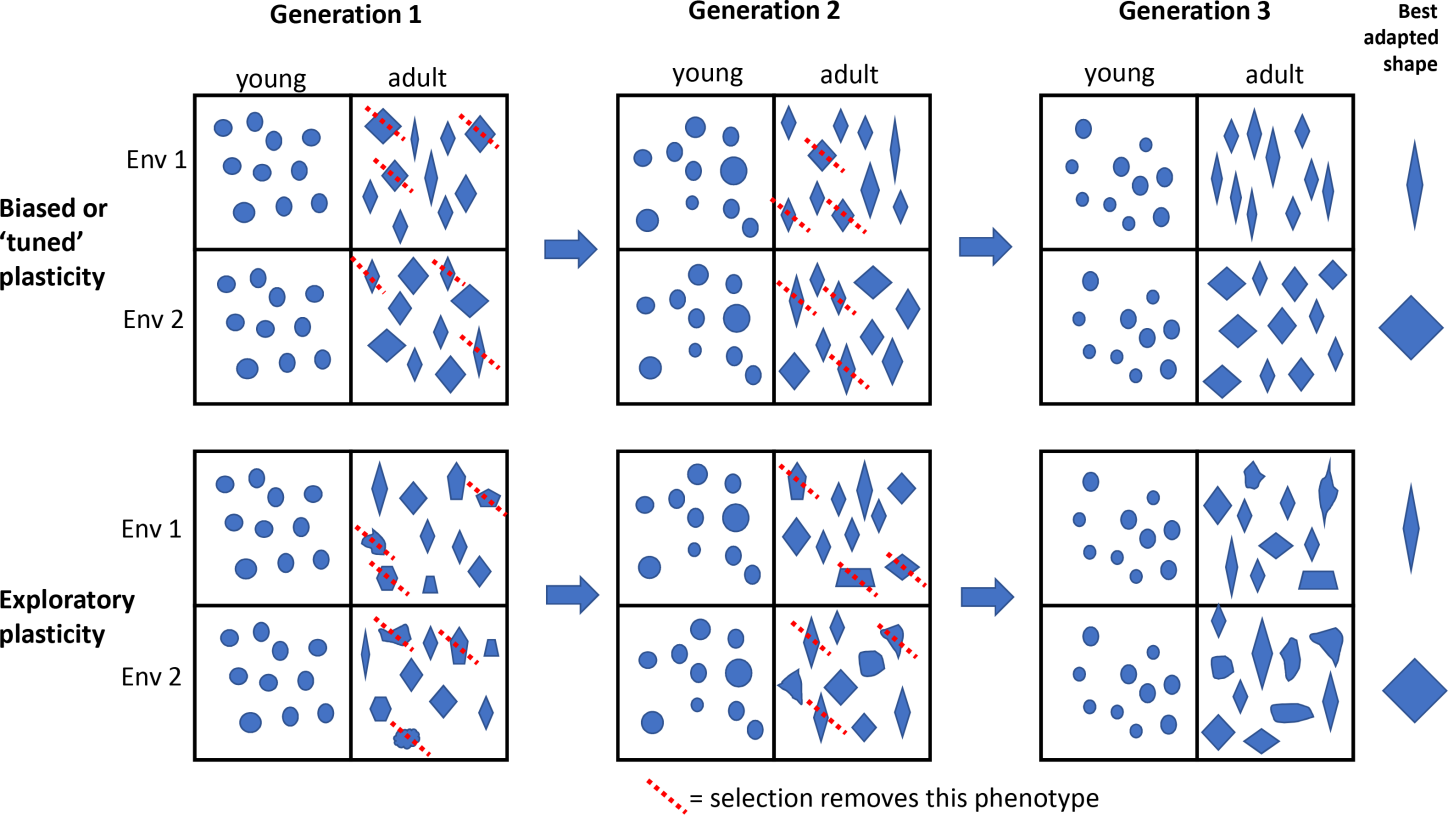
Plasticity-led evolution. Developmental plasticity refers to the way that an individual genotype interacts with its environment during development to produce a particular phenotype. According to the theory of plasticity-led evolution, these interactions can be biased or 'tuned' over generations so that embryos develop to consistently produce the right phenotypes (represented by shapes in this schematic figure) for the environments they might encounter. If plasticity is biased or tuned (top two rows), then adaptation to different environments is more directed by the types of variations available for selection, and 'well-adapted' phenotypes (represented by the shape on the right of each row) are relatively common. If plasticity is more exploratory (bottom two rows), then adaptation could take longer and/or the lineage might evolve alternative solutions to the same environmental challenge, and there are relatively few 'well-adapted' phenotypes for a given trait. The red dotted line means that selection removes that phenotype. Anole lizards are found on many islands in the Caribbean, with some phenotypic variations appearing repeatedly in lineages that have evolved independently on different islands. The plasticity of the skeleton of young lizards is thought to have a role in this repeated evolution, but Feiner et al. report that the plasticity is more exploratory than expected, so its role in the repeated evolution is still a mystery.

Now, in eLife, Nathalie Feiner, Illiam Jackson, Kirke Munch, Reinder Radersma and Tobias Uller at Lund University and the University of Tasmania report an example in which developmental plasticity does not in fact facilitate adaptation (Feiner et al., 2020). They tested their ideas on a group of Greater Antillean anoles (greenish iguanid lizards) that have become iconic examples of a concept called 'evolution on repeat'. This concept is illustrated by a series of observations over the past 15 years that documents how anoles colonized many different islands in the Caribbean and then evolved independently into similar ecomorphs (that is, into forms associated with specific habitats such as tree crowns, tree trunks, the ground or grasses; Losos, 2009; Mahler et al., 2013; Reynolds et al., 2020).

The question Feiner et al. wanted to answer was: has tuned plasticity facilitated the repeated evolution of specific morphologies in anoles? It is well known that bones are responsive to mechanical stress, so the researchers focused on the plasticity and evolution of the skeletal anatomy in lizards from different climbing or running habitats. Their sample was 259 male anoles from 95 species that were assigned to one of six ecomorph categories, and for each anole they measured 132 features that might respond to mechanical stress, including bone length, tissue thicknesses and the three-dimensional shape of the pectoral (shoulder) and pelvic bones. Feiner et al. chose to examine the shapes of the pectoral and pelvic bones, in addition to limb lengths, because they have more dimensions along which to measure responsiveness to mechanical stress, thus making evolutionary changes easier to detect.

First, they looked for evidence of ecomorphs that had evolved in parallel (that is, evidence of phenotypical trait variations appearing repeatedly in lineages that have evolved independently). Of the 53 pairs of ecomorphs in their sample only five pairs had skeletons that could have evolved in parallel, and only 14 had evolved a significantly new shape of any kind. Although the level of parallelism was higher than would be expected by chance, it was still fairly low, and there was no consistency in how each divergence happened. This unexpected result was compounded when Feiner et al. evaluated these anatomical changes in the context of the phylogenetic history of the anoles, which had been detailed by previous genetic analyses (see, for example, Poe et al., 2017). Once the influence of ancestral relationships was removed statistically, the skeletal adaptations seemed even more random.

To test whether developmental plasticity aligns with evolutionary divergence in this system, Feiner et al. raised baby anoles of two divergent species from Cuba in different caged habitats, and measured changes in their skeletal anatomy. In one habitat the baby anoles were forced to climb thin branches, while they had to walk on flat surfaces in the other. However, the changes observed in the experiments (changes primarily in the pectoral region for one species, and in the pelvic region for the other) differed from those found in wild populations (mainly changes in limb length). The observation of plastic responses from 'different' parts of the skeletal anatomy in response to the 'same' ecological pressures suggests that a range of skeletal adaptations are accessible for selection, perhaps as alternate solutions to the same problem.

Finally, Feiner et al. checked to see if the strongest plastic responses correlated with the best running or climbing improvement in the baby anoles. Was developmental plasticity tuned to actually help the lizards climb or run better in these new environmental challenges? Again, there was no correlation between plasticity and functional performance. This result means that environmental challenges did not induce useful plastic responses, and different but closely related species from the same lineage adapted their skeletons differently to the same habitat challenge.

The mechanical responsiveness of the skeleton has been proposed as a likely candidate for alignment between plasticity and evolutionary responses. However, while bone plasticity is significant in these lizards, it does not seem to help with performance nor does it help predict evolutionary trajectory over the long term. That said, developmental plasticity must be involved in evolution, since it is firmly tied up with the production of variation, but its exact role in the lives of animals and through the history of lineages is still a mystery.
